# The Socio-Cultural Construction of Menstruation in the Ghanaian Context: A Qualitative Study of the Perspectives of Parents, Teachers, and Adolescent Girls

**DOI:** 10.3390/ijerph22030349

**Published:** 2025-02-27

**Authors:** Sitsofe Gbogbo, Israel Wuresah, Emmanuel Gbogbo, Wisdom Kudzo Axame, Priscilla Klutse, Robert Kokou Dowou, Sarah Odi Mantey, Sarah Abena Yeome Ayitey, Ishmael Boateng, Paramount Eli Nelson, Nuworza Kugbey, Victor Christian Korley Doku, Julie Hennegan, Frank Baiden, Fred Newton Binka

**Affiliations:** 1 Fred N. Binka School of Public Health, University of Health and Allied Sciences, PMB 31 Ho, Ghana; iwuresah22pg@sph.uhas.edu.gh (I.W.); egbogbo0@gmail.com (E.G.); wisdomaxame@gmail.com (W.K.A.); klutsepriscilla94@gmail.com (P.K.); 2017rdowou@uhas.edu.gh (R.K.D.); maameabenamantey@gmail.com (S.O.M.); ayiteysarah40@gmail.com (S.A.Y.A.); ishmaelboateng@gmail.com (I.B.); paramountkwameeli@gmail.com (P.E.N.); fbaiden@uhas.edu.gh (F.B.); fred.binka@gmail.com (F.N.B.); 2 School of Natural and Environmental Sciences, University of Environment and Sustainable Development, PMB Somanya, Ghana; nkugbey@uesd.edu.gh; 3 Faculty of Life Sciences and Medicine, King’s College London, London WC2R 2LS, UK; victor.doku@kcl.ac.uk; 4 Maternal, Child and Adolescent Health Program, Burnet Institute, Melbourne, Victoria 3001, Australia; julie.hennegan@burnet.edu.au

**Keywords:** menstruation, taboo, belief, socio-cultural, menarche, Ghana

## Abstract

(1) Background: Menstruation, experienced by 1.8 billion girls and women globally, is often misunderstood and surrounded by taboos and misconceptions, particularly in low-income countries like Ghana. This study explores the Ghanaian socio-cultural representation of menstruation and the cultural experiences of young girls during menarche. (2) Methods: This qualitative exploratory study was conducted in five Senior High Schools in Ghana’s Volta Region, using purposive and convenience sampling. Fifteen Focus Group Discussions (FGDs) were conducted, including five FGDs each for female students, teachers, and parents, with 10–12 participants per group. All FGDs were audio-recorded, transcribed, translated, and imported into MAXQDA 2022 for thematic analysis. (3) Results: This study identified various socio-cultural beliefs and practices surrounding menstruation in Ghana, including the celebration of menarche, viewing menstrual blood as unclean, and cultural taboos like household, religious, and social restrictions. (4) Conclusion: Despite shifting beliefs and practices, cultural and social practices/restrictions regarding menstruation are persistent in the Volta Region of Ghana, and these practices often determine how society interacts with menstruating women. These practices often determine how society interacts with menstruating girls and women and hinder optimal menstrual health and hygiene.

## 1. Introduction

Menstruation is a natural physiological process experienced by 1.8 billion girls and pre-menopausal women globally and can be experienced about 300 times in a woman’s lifetime [1,2]. Rapid transformations due to the hormonal changes that accelerate physical, cognitive, and psychosocial growth usually occur during adolescence [3]. From the many changes during adolescence, the occurrence of menstruation or menarche is a natural event that is both a physiological and psychological milestone in a woman’s reproductive life [4]. Although this physiological occurrence is universal, the socio-cultural context profoundly shapes its meanings, practices, and the experiences of those who menstruate [5].

The social constructionist posits that menstruation is not merely a biological event but a phenomenon shaped by socio-cultural values and interpersonal relationships [6,7]. In Ghana and similar low- and middle-income contexts, this perspective reveals how menstruation is often perceived through the lens of cultural beliefs that frame it as impure or shameful [8,9]. Thus, menstruation remains a silent subject. This silence discourages open discussions within families and communities, reinforcing secrecy and stigma. The socialization process informs how these norms are constructed, as girls grow up learning that menstruation should be managed discreetly, with minimal acknowledgment in public or even within their own households [10,11]. As a result, cultural restrictions often limit girls’ participation in daily activities, religious practices, and social interactions during menstruation [12].

These cultural constructions are further reinforced by social narratives that label menstruation as taboo [11]. This affects how girls perceive menstruation and impacts their ability to seek support, access resources, and engage in conversations about menstrual health [8]. Also, gender dynamics and broader societal structures contribute to these limitations, demonstrating that even physiological experiences are shaped and interpreted through cultural and social contexts.

Socialization processes within families and communities play an important role in shaping attitudes toward menstruation as well. In many Ghanaian households, discussions about menstruation are limited, and adolescents often receive minimal information, leading them to internalize social expectations of silence and secrecy around menstruation. Through socialization, girls learn not only the practical aspects of managing menstruation but also culturally endorsed attitudes, such as viewing menstruation as a private matter or something to be hidden [13]. This layer of the social constructionist framework highlights how menstruation becomes a deeply internalized social construct influenced by generational teachings.

Gender dynamics are also pivotal in constructing menstruation as a socio-cultural phenomenon. In many societies, including Ghana, menstruation is embedded within broader gendered expectations about female behavior, purity, and propriety. Gendered norms often dictate that menstruating women maintain modesty and discretion, reinforcing ideas of contamination and exclusion. These dynamics perpetuate the belief that menstruation is a female-only matter, limiting access to support systems and resources for menstrual health.

**Figure 1:**
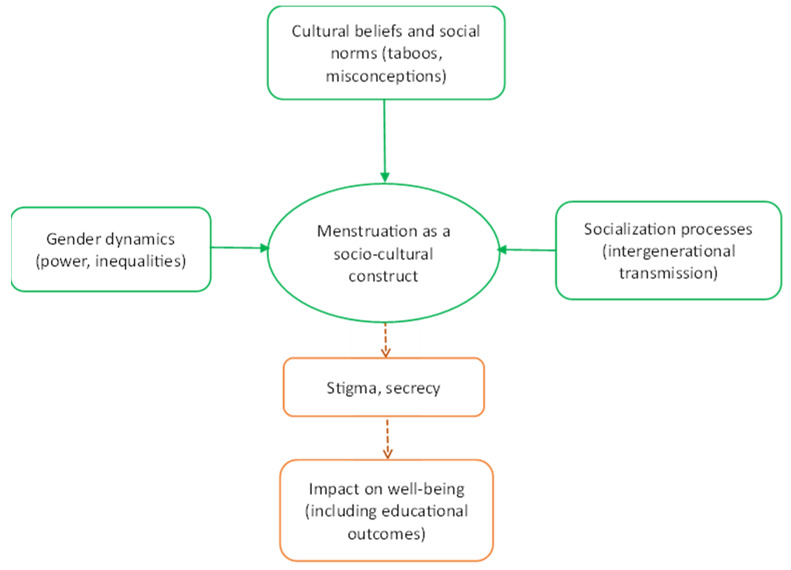
Social constructionist framework on menstruation in Ghana.

To explain how menstruation is viewed, our study adapts this framework and centers on cultural beliefs, social norms, socialization processes, and gender dynamics to provide rich qualitative data for understanding how menstruation is more than a biological event. These elements shape menstruation as a socio-cultural construct, influencing how girls manage, experience, and internalize their menstrual health. The framework (Figure 1) below presents the concept of this study.

The figure presents menstruation as shaped by cultural beliefs and norms, socialization, and gender dynamics, which are collectively thought to influence well-being of menstruators, including educational goals, through stigma and secrecy.

This study is particularly necessary because previous studies conducted in Ghana that explored menstrual restrictions were predominantly quantitative in nature and while surveys are important, they only tell the “what” [11,14,15], leading to a limited in-depth understanding of the cultural beliefs and practices shaping restrictions. Also, most studies in this area primarily focus on menstruating girls, making the inclusion of parents and teachers essential. In addition, the present study provides narratives from varied participants—parents, adolescent girls, and teachers—from a geographically broader perspective, supplementing the evidence from these past studies and building a strong argument for the development of targeted interventions. The findings from this study are important for designing targeted interventions, improving menstrual health and hygiene, and supporting the well-being and educational outcomes of girls in Ghana.

## 2. Methods

The reporting of this study follows the consolidated criteria for reporting qualitative research (COREQ) [16,17].

### 2.1. Setting

This study was conducted in five districts of the Volta Region of Ghana. These districts are Hohoe, Afadzato South, Kpando, Ho West, and Ho Municipality. The Volta Region is one of the 16 administrative regions in Ghana (West Africa). This region is located on the coast of the Gulf of Guinea and bordered by Togo. The Volta Region features a culturally diverse landscape, with various ethnic groups coexisting, each contributing unique traditions and beliefs that may shape perceptions and practices surrounding menstrual health and hygiene. The Ewe ethnic group is the dominant population, and this demographic pattern is reflected in the region’s high schools. Thus, the presence of multiple ethnic groups within the same geographic area offers a unique opportunity to examine how different cultural backgrounds within a shared social and educational context shape menstrual health and hygiene practices.

### 2.2. Study Design

This study was qualitative and adopted an explanatory study design. We employed this design because it helps in generating in-depth information on beliefs [18].

### 2.3. Study Population

Our target population for this study was adolescent girls who are students of major Senior High Schools in the five selected districts in the Volta Region. We also targeted their teachers and parents for participation in this study.

### 2.4. Sampling Procedure and Sample Size

Convenience and purposive sampling procedures were used for sampling all five Senior High Schools and participants. Specifically, the schools were selected using a convenience sampling approach, as they were the sites for the quantitative component of our project. Within these schools, adolescent female students who are menstruators and teachers who are involved in student well-being were purposively sampled. The selection of parents was based on convenience, ensuring accessibility during the study period. These approaches ensured variability among study participants. In total, we conducted 15 Focus Group Discussions (FGDs) across all five Senior High Schools, with 3 from each school. This comprised five FGDs for female students, five FGDs for teachers, and five FGDs for parents. For each of the FGDs, 10 to 12 participants were sampled to participate in the discussion.

### 2.5. Data Collection Instruments and Procedure

Data for this study were collected through face-to-face interactions in a group setting. All the female students and teachers’ discussions were conducted in English. However, the majority of parents’ discussions were carried out using native Ghanaian languages (Ewe and Twi). The choice of local language for the parents’ discussion was to ensure that we had in-depth information from them without any language barriers. To ensure that the use of the local languages did not affect the quality of the data collected, training was carried out. The research assistants received a two-day training session on qualitative research interviewing skills using a manual developed for this purpose. Each of the discussions with the participants lasted for about 60 min. The FGDs were conducted using FGD guides, which facilitated discussions about socio-cultural beliefs regarding menstruation and prevailing cultural and religious practices, and were piloted among similar populations to check for clarity and comprehension of the questions before the start of the data collection. Also, the guides were given to experts in the field of qualitative study for them to peruse in detail to check for the appropriateness of the questions. Audio recorders and hand-written notes were used to record the discussions. The use of both the audio recorders and hand-written notes was to ensure that the interview process was not halted if any of the equipment (pen or audio recorder) broke down during the interview process. After each interview, field notes were taken and referred to during the analysis, which included the participants’ nonverbal indications, worries, and interviewers’ reflections.

### 2.6. Data Processing and Analysis

All the recorded discussions from the participants were transcribed verbatim into a Microsoft Word document from which codes and themes were developed using flexible thematic analysis [19]. Transcriptions were carried out by trained transcribers with competencies in the local language. The transcripts were quality-checked by an independent person with both language and grammar competencies to ensure that the content of the transcripts was accurate. The transcripts were read and edited to resolve any omissions and mistakes in the original transcripts. Familiarization with the data was performed to take note of key ideas and recurrent themes. MAXQDA qualitative analysis software 2022 version was used to develop the codes and identify themes. The method employed involved the integration of pre-established codes derived from research questions, literature review, and newly generated codes that emerged from the data itself during the analysis phase. The coding process involved identifying and labeling ideas in each transcript. The coding process was informed by two primary considerations: deductive, where themes were preselected based on the guide used for the interview, and inductive. Before the coding process, the team had two separate meetings to agree on the constitution of a code and the labeling of emerging issues in the recorded audio. This was to ensure similar ideas occurring in any transcript analyzed by team members would be labeled or coded the same way. In any case of discrepancies, the views of an expert committee of the project were sought on what constituted a code. After the first round of coding, through constant comparison where observations from all transcripts were compared, a table was generated in Excel to align all the ideas in all transcripts against each other. From this table, dominant ideas and less dominant ideas were observed. The themes were defined and named, and a detailed analysis was conducted and written based on how they fit into the broader story of the data. To ensure the validity of the analysis, we used extracts from the data that capture the essence of each theme being demonstrated to develop the full write-up of the report. A frequency table was, however, used to present the socio-demographic characteristics of the participants.

## 3. Results

### 3.1. Background Information of Participants

Table 1 A–C present the background characteristics of the various groups that participated in this study.

### 3.2. Thematic Results

The analysis of the teachers, parents, and students’ interviews on the social-cultural construction of menstruation yielded two major overarching themes. One theme reflects the celebration that comes with adolescent girls’ menarche and the other theme indicates the taboos that are associated with menstruation in Ghanaian society. These major themes are supported by minor themes (see Table 2).

#### 3.2.1. Celebration of Menarche

Participants shared that a girl’s first menstruation is celebrated by the entire family, marking her transition into adulthood and womanhood. This celebration, as described by several participants, involves offering the adolescent two boiled eggs as part of a meaningful ritual. While the exact customs may vary across ethnic groups, the core symbolism remains: one egg is meant to be chewed, representing virtue and the girl’s initiation into womanhood, while the other is to be swallowed, symbolizing fertility and readiness for future motherhood. Participants noted that if the girl mistakenly chews the egg meant to be swallowed, it is believed that she has “chewed her babies”, symbolically jeopardizing her fertility. The following quotes capture participants’ perspectives on this tradition:

*“For our forefathers when you menstruate for the first time there is an event they celebrate for the person. They wear clothes and celebrate by boiling eggs and yams and mixing them. So, here when a girl menstruates for the first time her family celebrates her with boiled eggs to welcome her to womanhood”*

(Parent, 40 years, female).

*“For my daughter when she first saw her menses, she came running to me that she was discharging blood. So, I told her to clap for herself because she is now a woman. In Ewe [Majority Ethnic group in the Volta Region of Ghana] when you see your menses for the first time, you will be given an egg”*

(Parent, 55 years, female).

*“When I menstruated for the first time, my mother boiled two eggs for me and she said I should swallow one and I should chew one. She said the one that I will chew and swallow all has a meaning, if I chew it [egg], I have to swallow one before chewing it [egg], the one that I am swallowing is like virtue for me but if I try to chew the one that I have to swallow is going to be like I have chewed all the eggs in my womb but the one that I chewed after swallowing that one is like happiness in my family. And I was like okay but I found it difficult to swallow the egg because that was my first time”*

(Student, 16 years).

#### 3.2.2. Social Taboos Associated with Menstruation

Although the first occurrence of menstruation is often celebrated as a milestone symbolizing growth and transition into womanhood, menstruation as a whole remains encircled by numerous taboos. These stigmatizing practices and discriminatory attitudes stem from both community beliefs and family practices, manifesting in various restrictive and negative behaviors rooted in cultural misconceptions and prejudice about menstruation. Through a comparative presentation of findings, it emerged that these taboos include both household restrictions, which limit activities within the family setting, and communal restrictions, which impose boundaries on menstruating women in public and cultural spaces.

##### Household Restrictions

In many households, menstruating girls and women face limitations rooted in cultural beliefs. Some families enforce strict rules prohibiting them from cooking for men, including fathers, chiefs, and brothers, using communal utensils, or even occupying the same living spaces. Parents justify these restrictions as a way to uphold tradition and ensure spiritual purity within the household:

*“In my father’s house, anyone who is menstruating isn’t allowed to stay. If you ignore it, the gods will make your menses stop until a cleansing ritual is performed.”*

(Mother, 53 years)

Others describe specific isolation practices, such as separate rooms and designated household items:

*“According to tradition, when they build a house, a room is allocated for menstruating women. They occupy that space until their period ends before rejoining the household.”*

(Father, 53 years)

However, the perspectives on these restrictions vary. Some parents recognize these customs but choose not to enforce them:

*“I heard that if a man gives a woman a lift and later finds out she was menstruating, he must perform a cleansing ritual. But in my house, we don’t follow such beliefs.”*

(Mother, 46 years)

For adolescent girls, these household restrictions evoke mixed emotions. Some feel excluded, while others appreciate the temporary break from domestic chores:

*“On my father’s side, when I am menstruating, I don’t cook and use a separate cup and plate. I feel uncomfortable because it seems like I’m alone, and nobody talks to me.”*

(Student, 19 years)

*“I’m the only girl child in our house, so when I menstruate, I don’t have to do chores. I’m happy because my mother continues doing everything.”*

(Student, 19 years)

Teachers observe how these restrictions affect girls’ emotional well-being. Some recognize the need for cultural sensitivity while advocating for education to counteract stigma:

*“In some communities, menstruating women are forbidden from using communal water buckets because they are seen as unclean. This is one of the areas where education is needed.”*

(Teacher, 45 years, female)

##### Communal Restrictions

Beyond the household, communal restrictions dictate where menstruating women can go, including places of worship, rivers, and certain parts of the chief’s palace. These rules are upheld out of respect for cultural traditions and spiritual beliefs:

*“In some shrines, a menstruating woman must not enter because she is considered ritually unclean.”*

(Father, 77 years)

*“In my hometown, menstruating women are forbidden from going to the riverside. Someone else must fetch the water for them.”*

(Mother, 45 years)

*“Menstruating women cannot enter the stool room in the chief’s palace, as it is believed to desecrate the spiritual stool.”*

(Father, 75 years)

While some students accept these customs, others question their validity, especially when personal experience contradicts the supposed consequences:

*“They say menstruating women should not go to the river, but once, I was swimming when my period unexpectedly started. Nothing happened to me.”*

(Student, 18 years)

Teachers emphasize the psychological impact of these restrictions, with one recalling how such customs made her feel isolated in her own adolescence:

*“When I was growing up, menstruating women were not allowed in certain churches. They would question you at the door before letting you in. It became so psychological that I sometimes cried because they made me feel as if menstruation was a sin.”*

(Teacher, 32 years, female)

These communal restrictions, while intended to uphold cultural values, reinforce stigmas that can affect young girls’ confidence and participation in social life. Some educators stress the importance of shifting cultural narratives to recognize menstruation as a natural biological process rather than a marker of impurity:

*“At times, societal perceptions force menstruating girls into isolation, making them unproductive during that period. We need education at the cultural level to allow them to function normally in society.”*

(Teacher, 38 years, male)

## 4. Discussion

This qualitative study explores the socio-cultural construction of menstruation within Ghana, emphasizing how these beliefs influence the menstrual experiences of adolescent girls and women. Using a social constructionist lens, the findings reveal that menstruation in Ghana is far more than a biological event; it is a culturally embedded process, with distinct social meanings, rituals, and restrictions that impact daily life, education, and social participation for girls and women.

This study highlights how menarche, marking the onset of menstruation, symbolizes a significant life transition from childhood to womanhood within Ghanaian culture. This transition is celebrated through specific rituals, such as the offering of cooked eggs, reflecting values tied to virtue and familial happiness [20]. These symbolic acts, particularly within the Volta Region, illustrate the ways in which cultural practices reinforce social norms regarding female maturity, marriage readiness, and fertility ([21], [22] p. 201). The ritualistic swallowing and chewing of eggs as part of menarche ceremonies serve not only as a means of celebration but also as a form of socialization, teaching young girls the importance of fertility and familial expectations. In line with practices observed in other regions, such as India and parts of West Africa, this reflects a shared cultural reverence for the onset of menstruation.

Further, this study reveals that menstruating women and girls in Ghana often face restrictions based on beliefs about purity and contamination. These taboos manifest in various forms, such as prohibitions on preparing food for men or chiefs and restrictions on visiting rivers and participating in religious activities. These practices are seen as preventing men from being contaminated [23] and preventing contamination of water sources, an essential communal resource [24,25]. Through these restrictions, the social constructionist framework reveals how menstrual taboos sustain gendered hierarchies and reinforce the need for women to maintain purity, ultimately influencing their social standing and freedom within the community.

Interestingly, this study highlights a dual perspective on menstrual restrictions as indicated by both parents and adolescent girls. The present findings indicate flexibility in enforcing restrictions among some parents although they acknowledge their existence. Parents are essential catalysts for shaping negative beliefs. This dual perspective balances the importance of upholding tradition with the realities of modernity. While many families continue to disseminate cultural taboos, others recognize the need for change, particularly as increasing awareness and education challenge restrictive practices. Also, adolescent girls may report feelings of being excluded or stigmatized due to these restrictive practices while others experience a sense of relief, as menstrual restrictions exempt them from daily household responsibilities [24,26]. This ambivalence—where menstruation-related restrictions both marginalize and occasionally offer reprieve—illustrates a multifaceted negotiation between cultural expectations and individual coping strategies. As menstruating girls are temporarily exempt from certain chores, they may experience a break from physical labor, albeit within a framework that still reinforces their perceived impurity.

A critical issue that was not reported in this present study is the limited education about menstruation health and hygiene. Studies indicate that despite high awareness, gaps in comprehensive menstrual education remain, particularly in rural areas where social stigmas are stronger [11]. This lack of knowledge among adolescent girls, parents, and community members at large contributes to ongoing restrictive practices, such as isolating menstruating girls or preventing them from participating in religious and social activities [27]. Thus, concerted efforts are required to actively engage and educate adolescent girls, parents, and community members toward reshaping the socio-cultural lens through which menstruation is perceived.

In summary, menstruation in Ghana is shaped by gendered expectations and socialization. In many Ghanaian households, menstruation is treated as a private or even shameful matter, with limited discussion or education provided to adolescent girls. Socialization practices reinforce these attitudes, as families often perpetuate cultural taboos, instilling beliefs about menstrual impurity and secrecy [13]. As observed in this study, most narratives indicate an alignment to beliefs that existed in participants’ communities channeled down to the present generation. These beliefs create barriers for young girls, who may internalize these views and develop a limited understanding of menstruation as something to be concealed rather than accepted [11,25]. Also, the gendered framing of menstruation explains the social restrictions on menstruating women, positioning it as an exclusively female experience tied to modesty, silence, and control. This restricts the involvement of men and community members in menstrual education, ultimately limiting resources and support for menstruating girls.

This study’s findings suggest that socio-cultural beliefs around menstruation in Ghana have significant implications for menstruating girls. The taboos and restrictions surrounding menstruation not only limit women’s participation in daily activities but also contribute to an environment of stigma and misinformation [8,9].

## 5. Conclusions

This study highlights the socio-cultural construction of menstruation in Ghana, where beliefs, taboos, and practices around menstruation significantly influence the daily lives and social roles of adolescent girls and women. Menarche is celebrated as a transition to womanhood, yet menstruation as a whole remains encumbered by restrictive taboos. The findings suggest that restrictive beliefs reflect deep-seated notions of purity and impurity. Although cultural traditions offer some young women moments of rest from chores, they simultaneously reinforce social isolation and stigma, particularly where these customs are rigidly enforced. Recognizing that parents, teachers, and adolescent girls express varied levels of acceptance of these practices, this study reveals a need for culturally respectful interventions that balance tradition with modern insights to promote menstrual health and alleviate restrictive taboos.

To achieve this, educational materials should be developed to blend factual information with culturally relatable messaging for improved knowledge on menstruation. This study also recommends utilizing media and health promotion outreach to enhance menstrual health education and accessibility, especially in underserved areas. Radio and social media campaigns can effectively reach remote communities, sharing positive, accurate messages to dispel myths and normalize menstruation.

## 6. Strength and Weakness

The use of a qualitative approach in this study was critical for an in-depth understanding of the socio-cultural beliefs and practices surrounding menstruation in Ghana. Conducting the study in Senior High Schools and involving adolescents, parents, and teachers provided a broader insight that facilitates the application of the findings to the broader Ghanaian society.

However, the use of Focus Group Discussions as the data collection method could have introduced response and social desirability biases among the participants. These biases may have led the participants to report more socially acceptable practices or attitudes rather than their true perceptions. This potential limitation should be considered when interpreting the study’s findings and their transferability to the broader population.

## Figures and Tables

**Table 1:** (**A**): Adolescent schoolgirls. (**B**): Parents. (**C**): Teachers.

(**A**)		
**Variable**	**Frequency, N = 54**	**Percentage (%)**
Age		
15	5	9.3
16	14	25.9
17	24	44.4
18	9	16.7
19	2	3.7
Level of Education (Form)		
Form 1	17	31.5
Form 2	24	44.4
Form 3	13	24.1
Ethnicity		
Ewe	45	83.3
Akan	5	9.3
Ga/Dangbe	4	7.4
District		
Hohoe	10	18.5
Kpando	10	18.5
Ho West	13	24.1
Ho	11	20.4
Afadjato South	10	18.5
(**B**)		
**Variable**	**Frequency, N = 45**	**Percentage (%)**
Age		
25–34	5	11.1
35–44	16	35.6
45–54	12	26.7
55 and above	12	26.7
Sex		
Female	28	62.2
Male	17	37.8
Ethnicity		
Ewe	41	91.1
Akan	2	4.4
Hausa	2	4.4
District		
Hohoe	6	13.3
Kpando	9	20.0
Ho West	11	24.4
Ho	10	22.2
Afadjato South	9	20.0
(**C**)		
**Variable**	**Frequency, N = 49**	**Percentage (%)**
Age		
25–34	16	32.7
35–44	19	38.8
45 and above	14	28.6
Sex		
Female	16	32.4
Male	33	67.3
Ethnicity		
Ewe	46	93.9
Akan	2	4.1
Chamba	1	2.0
District		
Hohoe	7	14.3
Kpando	10	20.4
Ho West	11	22.5
Ho	10	20.4
Afadjato South	11	22.5

**Table 2:** Thematic table.

**Social Cultural Practices**		
Menarche Celebration	Offering of Boiled Eggs	
		Swallowing of eggs Eating (chewing) of egg
Taboos	Household Restrictions	
		Cannot cook for men (father, chiefs, brothers) Isolation (use different rooms and items) Not going into some rooms in the house [stool room]
	Communal Restrictions	
		Place of worship Riverside [cannot travel across river] Not allowed to go to some parts of chief’s palace

## Data Availability

All relevant data are within the manuscript. Any further requests regarding the data used for this study could be made through the corresponding author.
